# The improved properties and microstructure of β-solidify TiAl alloys by boron addition and multi steps forging process

**DOI:** 10.1038/s41598-019-47530-9

**Published:** 2019-08-27

**Authors:** Shulong Xiao, Yuyong Chen, Mingao Li, Lijuan Xu, Jing Tian, Dongdong Zhang, Jianhui Yang

**Affiliations:** 1 National Key Laboratory for Precision Hot Processing of Metals, Harbin Institute of Technology, Harbin, 150001 P.R. China; 2 School of Materials Science and Engineering, Harbin Institute of Technology, Harbin, 150001 P.R. China

**Keywords:** Aerospace engineering, Mechanical properties, Metals and alloys

## Abstract

A novel forging process has been designed for better mechanical properties of Ti-43Al-6Nb-1Mo-1Cr-(0,0.6)B alloys in this paper. Multi step forging process could provide much finer microstructure, higher room-temperature strength, increased high-temperature strength and elongation with these alloys. The forged alloys without boron exhibit strength and elongation as 676.05 ± 11.37 MPa and 41.32 ± 1.38% at 800 °C, while the average grain size represents as 12.63 ± 3.77 μm. The forged alloys with 0.6 at.% B represent better mechanical properties than the forged alloys without boron, due to the refined microstructure with dispersive borides. Meanwhile, the detailed mechanism of increased strength and elongation caused by finer microstructure were concluded and discussed.

## Introduction

TiAl alloys have obtained interests of researchers on aerospace applications due to light weight and high strength^1,2^. The insufficient mechanical properties^3,4^ decides the application and development of TiAl alloys. Therefore, the high Nb-TiAl alloys have been designed^5^ for mechanical properties improvement. There are also other methods to improve the mechanical properties of TiAl alloys, such as hot-forged, hot-rolled^6^ and alloying^7^. As β stabilizer, Mo^8–11^or Cr^12–14^ addition were effective ways to obtain the excellent oxidation resistance and creep strength. With Cr and Mo addition, a novel composition with Ti-43Al-6Nb-1Mo-1Cr(at.%) was designed for better mechanical properties.

The dynamic recrystallization (DRX) and dynamic recovery (DRV) would influence the mechanical properties of TiAl alloys after hot processing^15,16^. TiAl alloys manufacture through the conventional forming process has posed a challenge for technical applications. Especially, Nb, Mo and Cr addition could improve the mechanical properties of TiAl alloys. However, these refractory elements also increase the deformation resistance to hinder the hot processing^9,10^. Therefore, it is necessary to design an economic manufacturing process for these alloys to guarantee the excellent mechanical properties based on the recent research^17^.

Li^18^ *et al*. found that boron addition could promote the DRX behavior largely to soften the TiAl alloys in hot processing. In addition, boron addition could also improve the mechanical properties of conventional TiAl alloys applied in casting^19–22^. However, less researches referred to the effect of boron addition on the mechanical properties of forged TiAl alloys. Most β solidified TiAl alloys should also be forged or rolled for better properties or engineering application. Therefore, it is necessary to investigate the effect of boron addition on the properties and microstructure of forged TiAl alloys.

Two main objects focus on the economic manufacturing process and the effect of boron addition on the mechanical properties of forged Ti-43Al-6Nb-1Mo-1Cr alloys in this paper. Based on the recent study^18^, 0.6 at.% boron addition content could promote DRX formation largely, therefore, the boron content would be selected as 0.6 at.%.

## Experimental

**Figure 1:**
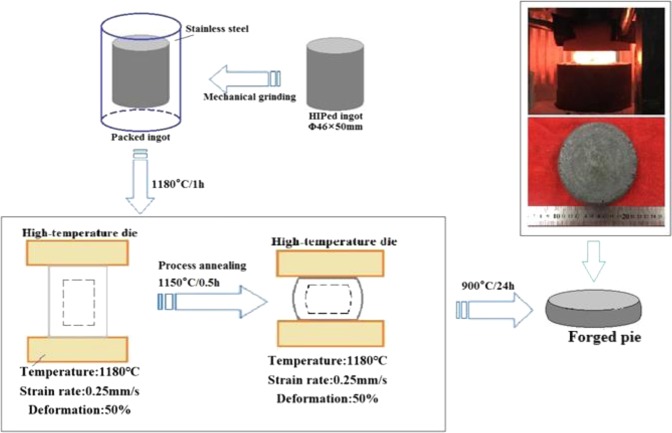
The schematic diagram of the multi step forging process of Ti-43Al-6Nb-1Mo-1Cr alloys.

Ti-43Al-6Nb-1Mo-1Cr-(0,0.6)B alloys were produced by means of induction skull melting (ISM), and then were hot isostatic pressed (HIPed) at 1200 °C for 4 h under a pressure of 140 MPa and aged at 900 °C for 24 h. The β solidified Ti-43Al-6Nb-1Mo-1Cr forged pie was obtained via multi steps forging process at 1180 °C. According to the deformation behavior and hot processing maps of Ti-43Al-6Nb-1Mo-1Cr alloys studied by Li^17^ *et al*., the processing could be designed as follow. The HIPed ingot (size: Φ46 × 50 mm) was cleaned through mechanical grinding and packed (via brazing) in 304 stainless steel. Afterwards, the packed ingot was preheated at 1180 °C for ~1 h prior to hot-forging and then subjected to multi-steps forging under the high-temperature dies. In the first step forging process, the deformation temperature was 1180 °C, the strain speed was 0.25 mm/s, the deformation rate was 50%. Before the second step forging, the forged ingot was heated at 1150 °C for 0.5 h during process annealing. The condition of the second forging process was same as the first step. After the multi steps forging process, the forged pie could be obtained with total deformation quantity 75%. Subsequently, the forged pie was tempered at 900 °C for 24 h and furnace-cooled to room temperature. The schematic diagram of the multi-steps forging process is shown in Fig. 1.

Li^18^ *et al*. found that 0.6 at.% boron addition could promote the DRXed grains nucleation largely to soften the alloys. Therefore, Ti-43Al-6Nb-1Mo-1Cr-0.6B alloys were selected for investigating the effect of boron addition on the mechanical properties of the forged alloys. Additionally, the process of Ti-43Al-6Nb-1Mo-1Cr-0.6B alloys is similar to the Ti-43Al-6Nb-1Mo-1Cr alloys in Fig. 1 while the forging process parameters could be obtained from the hot processing map^18^ as follow: the forging temperature is 1180 °C, the strain rate represents 0.01 s^−1^, the total deformation quantity is 75%, the forging heat preservation for 1 h, the process annealing for 0.5 h, the forged pie was tempered at 900 °C for 24 h and furnace-cooled to room temperature.

The tests were applied in Instron-5500 testing machine at romm temperature, 800 °C and 900 °C. The strain rate is 1 × 10^−4^ s^−1^. The microstructures were observed by scanning electron microscopy (SEM), electron back scattered diffraction (EBSD), transmission electron microscope (TEM), high resolution transmission electron microscope (HRTEM).

## Results and Discussion

### The microstructure evolution in the forged Ti-43Al-6Nb-1Mo-1Cr alloys

**Figure 2:**
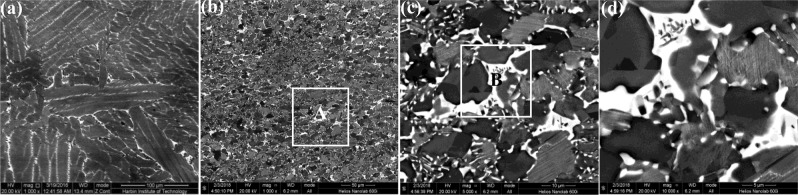
The casted and forged microstructure of Ti-43Al-6Nb-1Mo-1Cr alloys: (**a**) The casted microstructure^23^; (**b**) The forged microstructure; (**c**) The region A in (**b**,**d**) The region B in (**c**).

**Figure 3:**
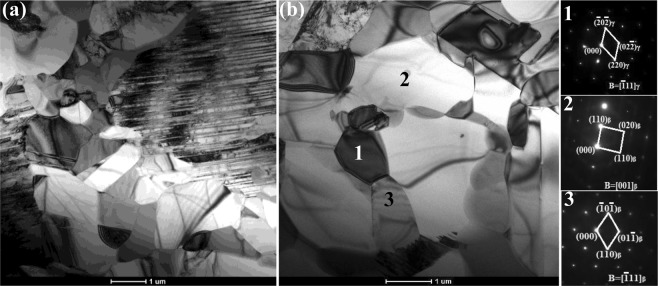
The bright-field TEM images of forged Ti-43Al-6Nb-1Mo-1Cr alloys: (**a**) The DRXed grains and (α_2_ + γ) lamellar colonies; (**b**) The DRXed grains and the corresponding selected area electron diffraction (SAED) patterns.

In Fig. 2, the microstructure of casted and forged Ti-43Al-6Nb-1Mo-1Cr alloys could be observed. In Fig. 2(a)^23^, columnar (α_2_ + γ) lamellar colonies form in the casted microstructure while the average colonies size is 584.94 ± 269.23 μm measured by OM images. The β/B2 phase distribute on the boundaries of columnar (α_2_ + γ) lamellar colonies. The low expansion microstructure and grains distribution of forged alloys are exhibited in Fig. 2(b,c). In Fig. 2(b), finer DRXed grains and (α_2_ + γ) lamellar colonies form perpendicular to compression direction while the average grain size is 12.63 ± 3.77 μm, as listed in Table 1. The grain sizes are decreased dramatically after multi-steps forging process. In Fig. 2(c), the equiaxed DRXed grains in alternating with finer (α_2_ + γ) lamellar colonies distribute uniformly and form a stable network structure. In Fig. 2(d), the needle shaped γ_0_ phase within β/B2 phase form on the (α_2_ + γ) lamellar colony boundaries. To ensure the DRXed phase, the corresponding selected area electron diffraction (SAED) patterns and the bright-field TEM images are exhibited in Fig. 3. It can be found that the DRXed β and γ phase appear in the forged microstructure while no orientation relationship occurs between the DRXed grains.

**Table 1:** The composition, grain sizes and mechanical properties of Ti-43Al-6Nb-1Mo-1Cr-(0,0.6) B alloys.

Alloys	Composition (at.%)	Grain sizes(μm)	UTS(MPa)			Elogation(%)		
			RT	800 °C	900 °C	RT	800 °C	900 °C
Casted T43	Ti-43Al-6Nb-1Mo-1Cr	584.94 ± 269.23	482.79 ± 9.21	534.85 ± 16.61	379.91 ± 4.30	0.13 ± 0.03	3.71 ± 0.33	15.65 ± 2.21
Forged T43		12.63 ± 3.77	645.65 ± 5.88	676.05 ± 11.37	383.16 ± 2.11	0.17 ± 0.01	41.32 ± 1.38	68.37 ± 4.71
Casted T43-0.6B	Ti-43Al-6Nb-1Mo-1Cr-0.6B	45.98 ± 27.23	623.67 ± 9.87	629.00 ± 0.78	506.69 ± 5.82	0.12 ± 0.16	3.51 ± 0.40	8.10 ± 0.57
Forged T43-0.6B		10.32 ± 4.16	843.51 ± 2.67	729.00 ± 0.51	387.98 ± 3.42	0.21 ± 0.12	68.03 ± 7.45	66.23 ± 3.91

**Figure 4:**
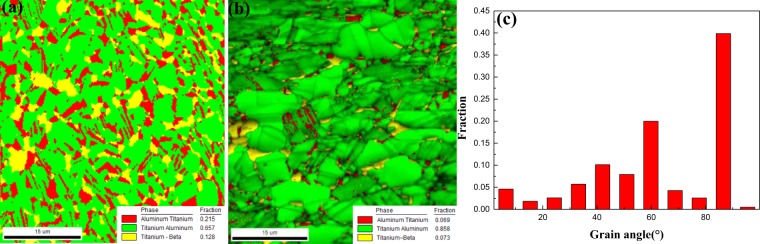
The EBSD images of forged Ti-43Al-6Nb-1Mo-1Cr alloys: (**a**) The phase distribution in the first step forged microstructure; (**b**) The phase distribution in the second step forged microstructure; (**c**) The statistical result about grain angles distribution in the second step forged microstructure.

In order to study the microstructure evolution during the multi-steps forging process, the EBSD image of the forged microstructure in Fig. 4. Compared to Fig. 4(a,b), with the steps increasing, the fraction of DRXed γ phase increases from 65.7% to 85.8% while the fraction of DRXed α_2_ phase decreases from 21.5% to 6.9%. The angles of grain boundaries distribution after second step process are listed in Fig. 4(c), the large angle grain boundaries occupy the fraction more than 95%, which represents that many DRXed grains occur. Meanwhile, the boundary angles 60° and 90° occupy large proportion. The DRXed grain boundaries would tend to 120° through necklace mechanism and boundaries migration^24,25^. When the angles were counted, 60° could be treated as 120° angle, because 60° is the other side of 120°. Additionally, the twinning and atoms rearrangement cause α_2_ phase parent lattice being reoriented by 90° around {10$\bar{1}2$} <10$\bar{1}\bar{1}$>^26,27^. Parts of α_2_ lamellae phase would deform, resolve or be reoriented between cylinder/base surfaces. Based on 90° rotation and the high stack fault energy, parts of DRXed α_2_ grains would grow along the [0001] in the reoriented α_2_ phase and form 90° boundaries with original α_2_ phase or the other matrix phase. The alloys were forged in (α + γ) phase region while many reoriented α grains formed along the [0001] with 90° boundaries during the thermal deformation. Afterwards, many γ phase nucleating from α grains would also exhibited same direction as matrix α grains with 90° boundaries during the cooling process. Therefore, during the forging process, the boundary angles 60° and 90° between the DRXed grains and remaining α_2_/γ lamellae formed normally.

**Figure 5:**
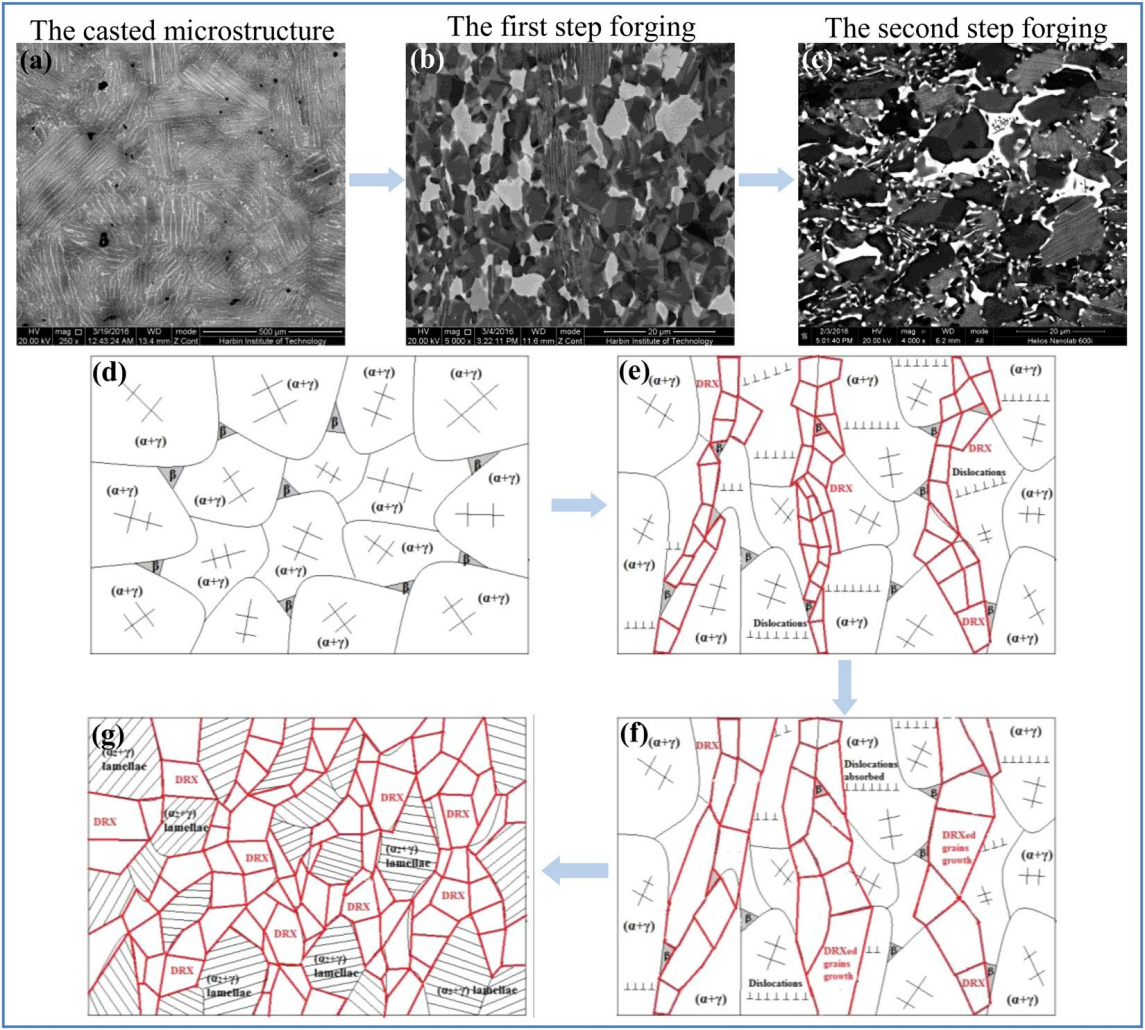
The microstructure evolution of Ti-43Al-6Nb-1Mo-1Cr alloys during multi steps forging process: (**a**) The as-cast microstructure; (**b**) The forged microstructure after the first step forging process; (**c**) The forged microstructure after the second step forging process; (**d**) The as-cast microstructure during insulation process before forged; (**e**) The DRXed grains nucleation and (α_2_ + γ) lamellae deformation during the first step forging process; (**f**) The DRXed grains growth during process annealing; (**g**) The DRXed grains and (α_2_ + γ) lamellae broken during the second step forging process.

The microstructure evolution during the multi-steps forging process could be concluded in Fig. 5. When Ti-43Al-6Nb-1Mo-1Cr alloys were heated at 1180 °C, the γ → α transformation occurs and the alloys stay in (α + γ) phase region. The microstructure of alloys would consist of β + (α + γ) phase, as shown in Fig. 5(a,d). The DRX behavior of γ phase dominates hot deformation^28,29^ while much DRXed α and γ phase would nucleate^17^. The β, α and γ phase represent A2, D0_19_ and L1_0_ structure at 1180 °C. The order of the slip systems quantity is β > γ > α^30–33^. The microstructure evolution in the first step forging process could be obtained in previous work^18^. When the alloys deform, dislocations slip through the softer β and γ phase and pile-up on the α phase surfaces. Because of four slip systems opening in γ phase, less dislocations pile-up in β phase, which leads to less DRXed β phase nucleation. Due to hard α obstacles, dislocations pile-ups cause much DRXed γ phase nucleation. Afterwards, parts of dislocations slip into α phase and pile-up while DRXed α phase nucleates. Therefore, much DRXed γ phase and less DRXed α phase form in the first step forged microstructure, as shown in Fig. 5(b,e).

The process annealing would be applied at 1150 °C for 0.5 h. The dislocation movement would be promoted to release stress concentration and to provide enough time for DRXed grains growth. The DRXed grains would grow and absorb dislocations, as shown in Fig. 5(f). In the second step forging process, the α phase would also be obstacles for dislocation movement while dislocations would pile up in the γ phase in front of α phase. The increased dislocation blocks in γ phase promotes the DRXed γ phase nucleation. Meanwhile, a large number of previous DRXed γ are broken to be tiny and equiaxed DRXed γ. Therefore, the content of DRXed γ phase would be further increased with the steps increasing below 1200 °C. The equiaxed DRXed grains in alternating with finer (α_2_ + γ) lamellar colonies distribute uniformly and form a stable network shaped structure, as exhibited in Fig. 5(g).

### The microstructure evolution in the forged Ti-43Al-6Nb-1Mo-1Cr-0.6B alloys

**Figure 6:**
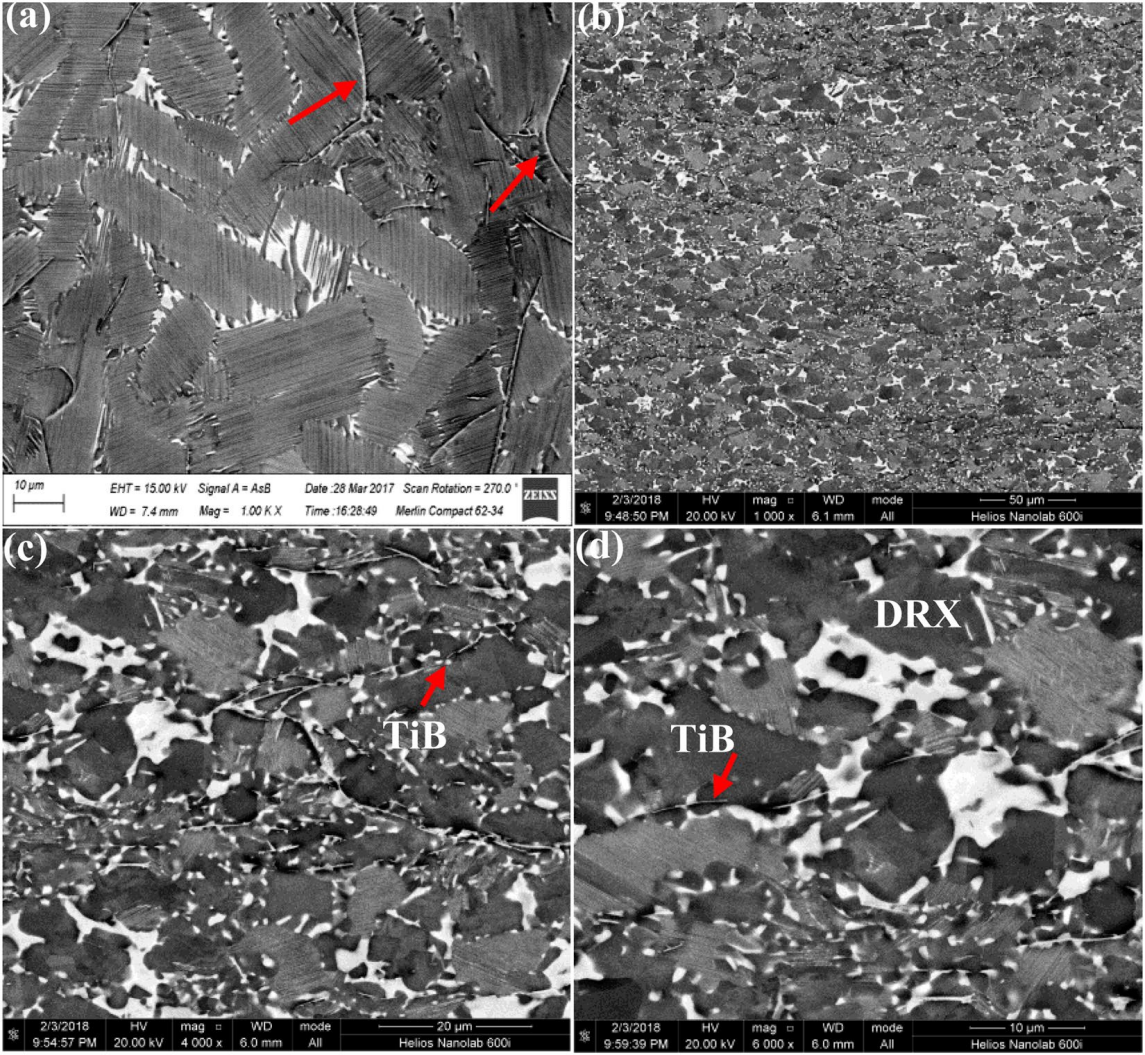
The cast and forged microstructure of Ti-43Al-6Nb-1Mo-1Cr-0.6B alloys: (**a**) The casted microstructure^34^; (**b**) Macroscopic forged microstructure; (**c**,**d**) DRXed grains, TiB and lamellar colonies.

To compare the effect of boron addition on the forged microstructure, the casted and forged microstructure of Ti-43Al-6Nb-1Mo-1Cr-0.6B alloys were observed in Fig. 6. The casted microstructure in previous work is exhibited in Fig. 6(a)^34^, with 0.6 at.% boron addition, the average sizes of (α_2_ + γ) lamellar colonies decrease, the β segregation disappear and stripe shaped borides appear, compared to Fig. 2(a). The forged microstructure under large expansion is obtained in Fig. 6(b), with the SAED patterns in ref.^34^, broken TiB distribute perpendicular to compression direction. TiB obstacles hinder dislocation movement to promote the DRXed grains nucleation, as shown in Fig. 6(c). The broken TiB and equiaxed DRXed grains in alternating with finer (α_2_ + γ) lamellar colonies distribute uniformly and form a stable network shaped structure in Fig. 6(d).

#### The as-cast microstructure evolution

**Figure 7:**
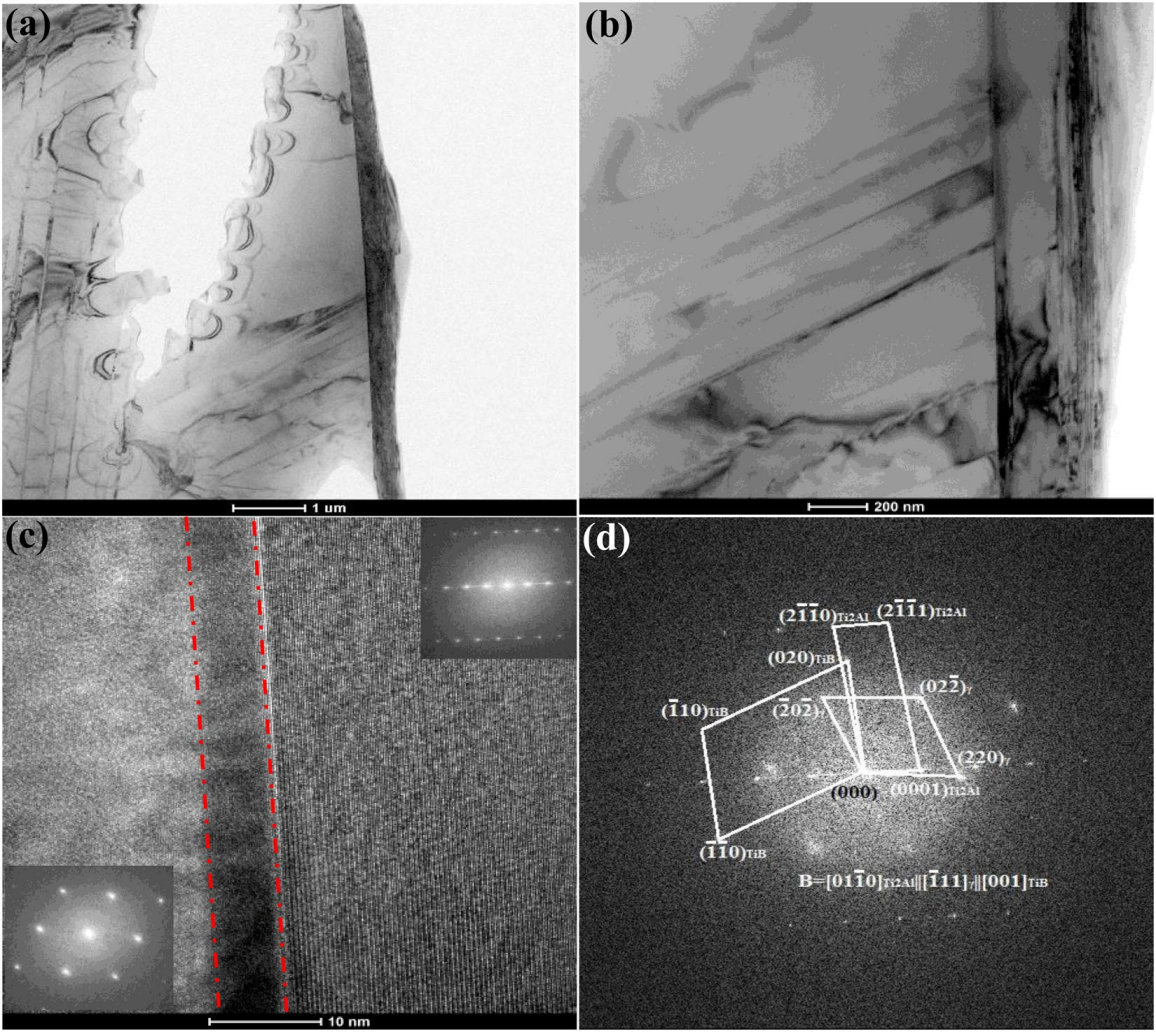
The microstructure of casted Ti-43Al-6Nb-1Mo-1Cr-0.6B alloys: (**a**) The bright-field TEM image of Ti_2_Al, γ and TiB phase^34^; (**b**) Detailed image of (**a**); (**c**) HRTEM image of interface; (**d**) SAED patterns.

In the casted microstructure of Ti-43Al-6Nb-1Mo-1Cr-0.6B alloys, Ti_2_Al phase could be observed on the interface of TiB and γ phase, as shown in Fig. 7. In Fig. 7(a,b)^34^, the TEM image of TiB and γ phase are exhibited, combining with Fig. 7(d), the orientation relationship of Ti_2_Al, TiB and γ phase could be ensured as [01$\bar{1}$0]_Ti2Al_||[$\bar{1}$11]_γ_||[001]_TiB_. By the chemical quantitative analysis of TEM, the ratio of Ti:Al:Nb:Mo:Cr:B on the interface could be ensured as 1.92:0.96:0.04:0.04:0.04:0.5. The interface phase is Ti_2_Al (P6_3_/mmc) and lattice parameters are a = 0.304 nm and c = 1.369 nm. In Fig. 7(c), the Ti_2_Al on the interface could be observed by HRTEM image.

In ref.^35^, TiB exhibit the orientation relationship (100)[0$\bar{1}$0]_TiB_||(11$\bar{2}$)[1$\bar{1}$0]_γ_ with neighbouring γ-matrix. Cao^36^ *et al*. found the orientation relationships between α_2_-Ti_3_Al, γ-TiAl and Ti_2_Al as (0001)[11$\bar{2}$0]_α2_||(0001)[11$\bar{2}$0]_Ti2Al_ ||(111)[0$\bar{1}$1]_γ_. Other researcher reported orientation relationships as [11$\bar{2}$0]_α2_||[011]_γ_||[11$\bar{2}$0]_Ti2Al_, (0001)_α2_||(11$\bar{1}$)_γ_||(0001)_Ti3Al_, (01$\bar{1}$0) _Ti2Al_||(01$\bar{1}$0)_Ti3Al_. Compared with the orientation relationship of Ti_2_Al, TiB and γ phase in these references, tiny angle between the habit plane occur in this paper. The angle between (2$\bar{1}\bar{1}$0)_Ti2Al_ and ($\bar{2}$0$\bar{2}$)_γ_ is 4.5°, while the angle between (020)_TiB_ and ($\bar{2}$0$\bar{2}$)_γ_ represents 16.3°. In comparison, boron addition causes TiB and Ti_2_Al formation and the angle between the crystal faces. This should be discussed to clarify the effect of Ti_2_Al formation on the forging process and thermal deformation of alloys.

(1) Transformation between Ti_2_Al and γ phase

He^37^ found many Ti_2_Al combining with α_2_ separate from supersaturated γ in Ti-51.7Al alloys, which were quenched at 1000 °C for 100 h. Ti_2_Al phase was thought as the transitional phase between α_2_ and γ phase while interfaces of Ti_2_Al and γ were parallel to {111}_γ_ or (0001)_Ti2Al_. The misfit dislocations with 4.2 nm thickness occur on the Ti_2_Al interface, in which a semi-atomic surface parallel to {111}_γ_ occurs and would not form stacking sequence along the interface. The lattice constants of γ phase represent a = b = 0.3976 nm, c = 0.4049 nm. Due to different lattice constants and crystal structure, the lattice misfit would form between Ti_2_Al and γ while the mismatch degree along particular direction can be calculated as follow:

(i) when the crystal faces are parallel to each other,
  1 $${\rm{\delta }}=\frac{2|{{\rm{d}}}_{1}-{{\rm{d}}}_{2}|}{{{\rm{d}}}_{1}+{{\rm{d}}}_{2}}\times 100 \% $$
  d_1_, d_2_ are the interfacial spacing of parallel planes.
(ii) when the angle occurs between the crystal planes,

2 $${\rm{\delta }}=\frac{1}{3}\mathop{\sum }\limits_{{\rm{i}}=1}^{3}\frac{|{{\rm{d}}}_{{\rm{s}}}^{{\rm{i}}}\,\cos \,{\rm{\theta }}-{{\rm{d}}}_{{\rm{n}}}^{{\rm{i}}}|}{{{\rm{d}}}_{{\rm{n}}}^{{\rm{i}}}}\times 100 \% $$

${{\rm{d}}}_{{\rm{s}}}^{{\rm{i}}}$, ${{\rm{d}}}_{{\rm{n}}}^{{\rm{i}}}$ are the interfacial spacing of parallel planes, θ is the angle between the crystal faces.

From the formula (1), the mismatch degree between Ti_2_Al and γ phase along [011], [101] and [110] could be obtained in Table 2. The mismatch degree between Ti_2_Al and γ along [110] is obviously much larger than these along the direction [101] and [011]. Influenced by lattice distortion, the crystal growth of Ti_2_Al phase along [110] would be impeded and the crystal growth rate along other directions are much faster. Finally, Ti_2_Al would form lamellae on the interfaces of γ and α_2_ phase. Ti_2_Al and γ phase grow as the habit plane (0001) and along [110] direction, which corresponds to (0001)[11$\bar{2}$0]_Ti2Al_|| (111)[0$\bar{1}$1]_γ-TiAl_ in references.

**Table 2:** Lattice misfits along different direction between the γ phase and Ti_2_Alphase.

Lattice direction	Lattice misfit (%)
[011]	10.55
[101]	10.55
[110]	>100

(2) Transformation between Ti_2_Al and α/α_2_ phase

The α_2_/γ lamellar structure would form in the primary α with A3 structure, while γ separate out with α_2_ during the cooling, the phase transformation is α → α_2_ → α_2_ + γ or α → α + γ → α_2_ + γ. The change of stacking fault sequence and composition provides the composition basis for γ phase nucleation. The interface existence of two atoms layers with b = 1/6 < 112 > Burgers vector mainly depends on the partial Shockley dislocation. The α_2_ and γ phase transformation mainly depends on the atomic stacking sequence change under element diffusion and Shockley dislocation. α_2_ phase is P6_3_/mmc structure with a = b = 0.5793 nm and c = 0.4649 nm and similar to Ti_2_Al structure. The instable Ti_2_Al is the transitional phase between α_2_ and γ phase. The essence of Ti_2_Al → α_2_ is a complete dislocation decomposing into incomplete dislocations. The incomplete Shockley dislocation starting changes the stacking sequence regular of dense stacking surfaces and cause phase transformation. However, α_2_ is dense-hexagonal structure, in which only dense plane (0001)α_2_ occur. Therefore, incomplete Shockley dislocation would only start on the plane (0001)α_2_. The stacking model of α_2_ phase along <11$\bar{2}$0> is…ABAB…, while the stacking model of Ti_2_Al phase along <11$\bar{2}$0> is …ABA’BA’CA….

**Figure 8:**
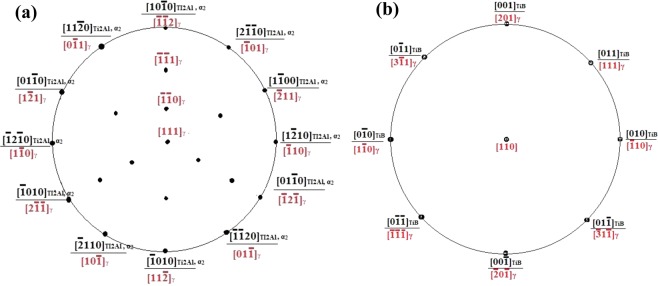
The projection polar graph: (**a**)α_2_, Ti_2_Al and γ phase on the (0001)α_2_ and (111)_Ti2Al_; (**b**) TiB and γ on the (100)_TiB_ and (11$\bar{2}$)_γ_.

The phase transformation between α_2_ and Ti_2_Al results from the stacking sequence variety caused by the dislocation movement. The lattice constant would change, but the crystal structure, the habit plane and direction of crystal growth would not change. It corresponds to the orientation relationship between α_2_ and Ti_2_Al by TEM. Figure 8(a) shows the projection polar graph of (0001)α_2_ and (111)_Ti2Al_, in which α_2_, Ti_2_Al and γ phase exhibit strict orientation relationship in crystal face and lattice constant.

(3) The phase transformation between γ and TiB

Based on recent references, TiB and γ in the casted TiAl alloys exhibited orientation relationship as (100)[0$\bar{1}$0]_TiB_||(11$\bar{2}$)[1$\bar{1}$0]_γ_. According to formula (1), the mismatch degree between two parallel (100)_TiB_ and (11$\bar{2}$)_γ_ along direction <1$\bar{1}$0> _γ_ could be calculated as 0.51%, while these two crystal faces combine as semicoherent interfaces. The growth rate of TiB along the direction <1$\bar{1}$0> _γ_ is much faster than other directions. In Fig. 8(b), the projection polar graph of TiB and γ on the faces (100)_TiB_ and (11$\bar{2}$)_γ_ could be calculated, in which the strict orientation relationship of TiB and γ phase occurs.

With TiB phase formation, the growth directions of Ti_2_Al and γ change while these phase maintain the orientation relationship [01$\bar{1}$0]Ti_2_Al||[$\bar{1}$11]γ||[001]TiB. However, tiny angles between <01$\bar{1}$0> _Ti2Al_ and (2$\bar{1}\bar{1}$0)_Ti2Al_, (020)_TiB_ and ($\bar{2}$0$\bar{2}$)_γ_ would occur due to boron addition. The angle between (2$\bar{1}\bar{1}$0)_Ti2Al_ and ($\bar{2}$0$\bar{2}$)_γ_ is 4.5° while the angle between (020)_TiB_ and ($\bar{2}$0$\bar{2}$)_γ_ is 16.3°. With boron addition, the solidification way of Ti-43Al-6Nb-1Mo-1Cr alloys changes to L → β + primary TiB → (α + secondary TiB) + primary TiB → ((α_2_ + γ + Ti_2_Al) + secondary TiB) + primary TiB. Ti_2_Al phase forms from atomic stacking faults variety in α → γ and γ → α_2_ transformation. Therefore, Ti_2_Al phase transforms from γ phase. The boron solution and TiB formation would cause the angle between crystal faces (2$\bar{1}\bar{1}$0)_Ti2Al_, (020)_TiB_ and ($\bar{2}$0$\bar{2}$)_γ_ during solidification. According to formula (2), the mismatch degree between crystal faces (2$\bar{1}\bar{1}$0)_Ti2Al_ and ($\bar{2}$0$\bar{2}$)_γ_ along the direction <01$\bar{1}$0> _Ti2Al_ is 5.97%. Meanwhile, the interplanar spacing of (020)_TiB_ and ($\bar{2}\,$0$\bar{2}$)_γ_ along the direction <001> _TiB_ are calculated as 0.305 nm and 0.143 nm, while the structure of these two crystal faces are semicoherent interface. After the angle 16.3° substituted in formula (2), the mismatch degree between these two faces is 2.36%. With the angle forming, the mismatch degree between (2$\bar{1}\bar{1}$0)_Ti2Al_ and ($\bar{2}$0$\bar{2}$)_γ_ decreases and the atom layer on the interface would combines much better. Meanwhile, with Ti_2_Al formation on the interface of TiB and γ, the (020)_TiB_ and ($\bar{2}$0$\bar{2}$)_γ_ crystal faces tilt to decrease the mismatch degree, even negligible. It indicates that Ti_2_Al formation could promote the combination of TiB and γ and increase anchoring strength of the interface.

Li^18^ *et al*. found that TiB obstacles cause dislocation piling-ups to promote DRXed grains nucleation. When the alloys are compressed, dislocation movement would change the atomic stacking sequence of Ti_2_Al and promote Ti_2_Al phase to transform to α_2_ or γ phase. The Ti_2_Al could provide the DRXed α_2_ and γ phase nucleation with chemical foundation. On the other hand, Ti_2_Al phase strengthens the combination of TiB and γ, which would hinder dislocation slipping better to promote the DRX formation. Therefore, the conclusion that Ti_2_Al promotes the DRXed α_2_ and γ phase nucleation could be ensured in this section.

#### The forged microstructure evolution

**Figure 9:**
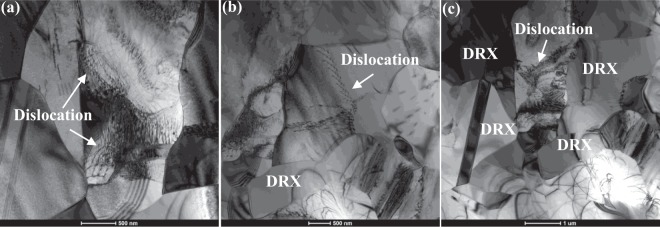
The bright-field TEM images of forged Ti-43Al-6Nb-1Mo-1Cr-0.6B alloys: (**a**,**b**) Dislocation movements into DRXed grains; (**c**) The DRXed grains obstacles for dislocation movements.

Many dislocations could be found in the forged microstructure of Ti-43Al-6Nb-1Mo-1Cr-0.6B alloys, as shown in Fig. 9. In Fig. 9(a,b), dislocations pile up on the DRXed grain boundaries, which indicates DRXed grains growth absorbing dislocation block. This absorbing process would cause the dislocations continually slipping into DRXed grains. In Fig. 9(c), dislocation pile-up on the boundaries of DRXed grains. It means that isotropic DRXed grains would hinder dislocation movement to promote the DRXed grains nucleation during the second step forging process.

**Figure 10:**
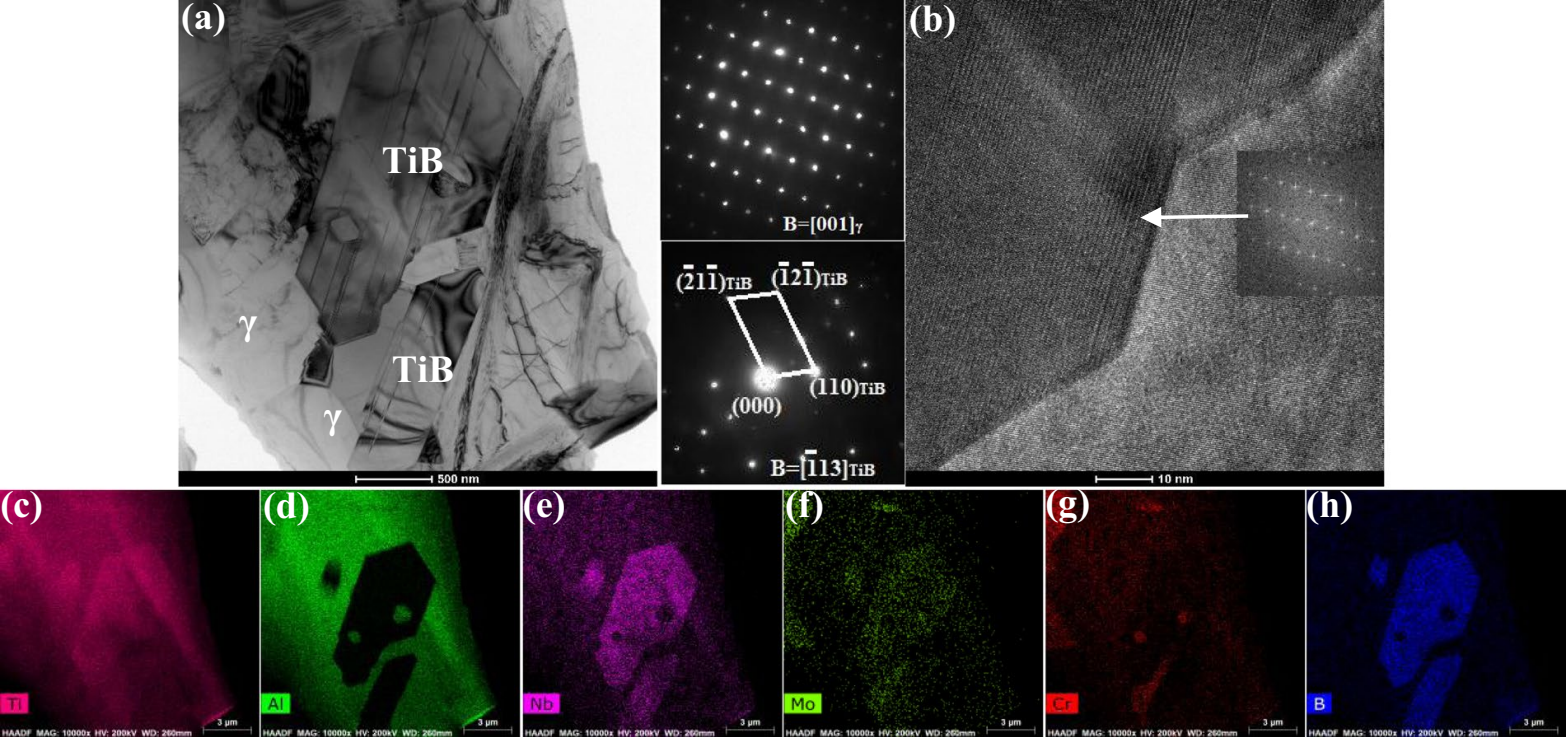
The TEM images of broken TiB in the forged Ti-43Al-6Nb-1Mo-1Cr-0.6B alloys: (**a**) The bright-field image of the broken TiB; (**b**) The HRTEM image of TiB, γ and interfaces; (**c**) Ti content; (**d**) Al content; (**e**) Nb content; (**f**) Mo content; (**g**) Cr content; (**h**) B content.

To ensure the structure and composition variety of TiB after forging process, the broken TiB is analyzed by TEM in Fig. 10. In Fig. 10(a), the stripe shaped TiB is broken, the matrix γ fills into the gap of broken TiB. According to SAED patterns and the chemical composition maps, the crystal structure and composition could be ensured as TiB and γ phase while the Ti_2_Al in casted microstructure disappear after forging. In Fig. 10(b), the HRTEM image of TiB, γ and interfaces could be seen. No dislocations but many Nb and Mo atoms solid solution occur in the TiB. It indicates that dislocations would slip into TiB but dislocation block would cause stress concentration to break TiB.

**Figure 11:**
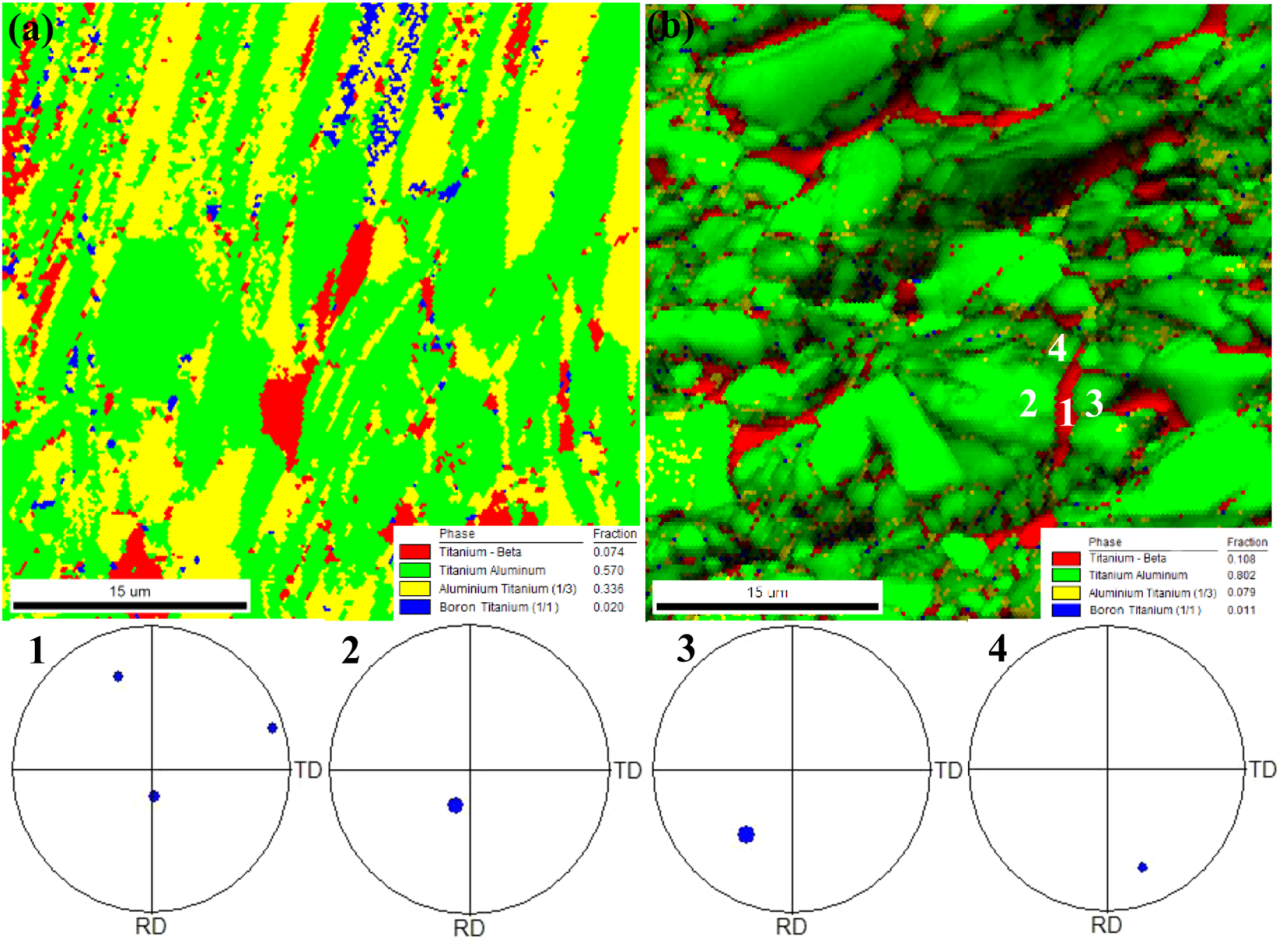
The EBSD images of forged Ti-43Al-6Nb-1Mo-1Cr-0.6B alloys: (**a**) The phase distribution in the (**a**) first step forged microstructure; (**b**) second step forged microstructure.

To compare the phase transformation, the EBSD images of Ti-43Al-6Nb-1Mo-1Cr-0.6B alloys in different steps forging process are exhibited in Fig. 11. With forging steps increasing, the content of DRXed γ phase increase from 57.0% to 80.2% while the DRXed α_2_ phase content decrease from 33.6% to 7.9%. Due to forging steps increasing, the TiB are broken again and again which causes the sizes of TiB decrease. The tiny broken TiB are difficult to measured, therefore, the content of TiB (1.1%) in the second step forged microstructure is much less than that (2.0%) in the first step forged microstructure. In Fig. 11(b), the pole maps of point 1,2,3 and 4 indicate that the DRXed γ, β and α_2_ exhibit no orientation relationship.

The microstructure evolution of Ti-43Al-6Nb-1Mo-1Cr-0.6B alloys during the forging process could be concluded. The alloys stay in (α + γ) phase and the β content maintains stability at 1180 °C^38,39^. The microstructure evolution in the first step forging process could be obtained in previous work^18^. The order of the slip systems quantity is β > γ > α > TiB^30–33^. When the alloys deform, less dislocations pile-up in β phase due to many slip systems opening in γ and β phase. This causes less DRXed β phase nucleation. Much DRXed γ phase nucleate, because many dislocations pile-up in γ that in front of α and TiB obstacles. Afterwards, parts of dislocations slip into α phase and pile-up in front of TiB surfaces. Then less DRXed α phase would nucleate. Therefore, much DRXed γ phase and less DRXed α phase form along the broken TiB in the first step forged microstructure, as shown in Fig. 11(a).

During the process annealing, the DRXed α and γ phase absorb the dislocations and grow along the broken TiB. In the next step forging process, the DRXed α, γ phase and broken TiB are broken are broken again. The α phase would also be obstacles for dislocation movements. Dislocation pile-ups in the γ phase in front of the α surfaces would promote the DRXed γ nucleation and increase the DRXed γ phase content. Therefore, the content of the DRXed γ phase in the second step forging process is increased dramatically.

### The mechanical properties of the forged Ti-43Al-6Nb-1Mo-1Cr-(0,0.6) B alloys

**Figure 12:**
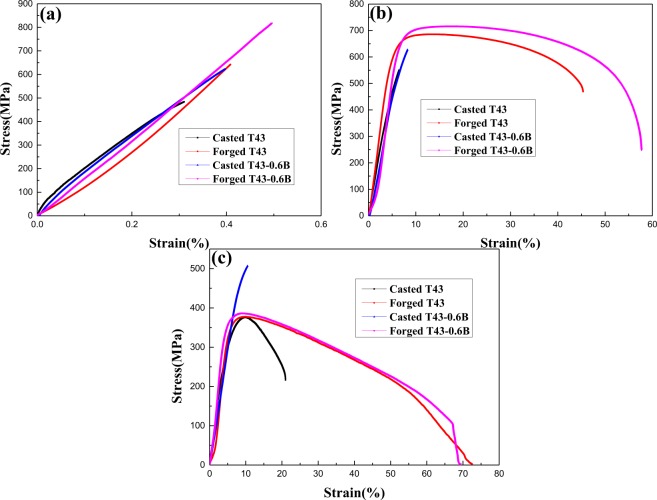
The tensile curves of as-cast and as-forged Ti-43Al-6Nb-1Mo-1Cr-(0,0.6) B alloys at: (**a**) Room temperature; (**b**) 800 °C; (**c**) 900 °C.

The tensile curves of casted and forged Ti-43Al-6Nb-1Mo-1Cr-(0,0.6)B alloys are exhibited in Fig. 12 while the detailed tensile properties are listed in Table 1. It can be found that the forged Ti-43Al-6Nb-1Mo-1Cr-0.6B alloys exhibit larger UTS and elongation than the forged alloys without boron containing, which means boron addition could improve better mechanical properties of forged alloys.

In Fig. 12(a,b), it can be seen that the forged alloys exhibit the dramatically increased UTS and elongation, compared to the casted alloys at room temperature and 800 °C. In Fig. 12(b), the forged Ti-43Al-6Nb-1Mo-1Cr-(0,0.6)B alloys display higher UTS and elongation than the casted Ti-43Al-6Nb-1Mo-1Cr alloys at 900 °C. However, the UTS of all the forged alloys are lower than casted Ti-43Al-6Nb-1Mo-1Cr-0.6B alloys at 900 °C. The UTS and elongation variety should be explained to investigate the effect of multi steps forging process and boron addition on the mechanical properties at different temperatures.

### The microstructure evolution of the stain region on the tensile specimens

**Figure 13:**
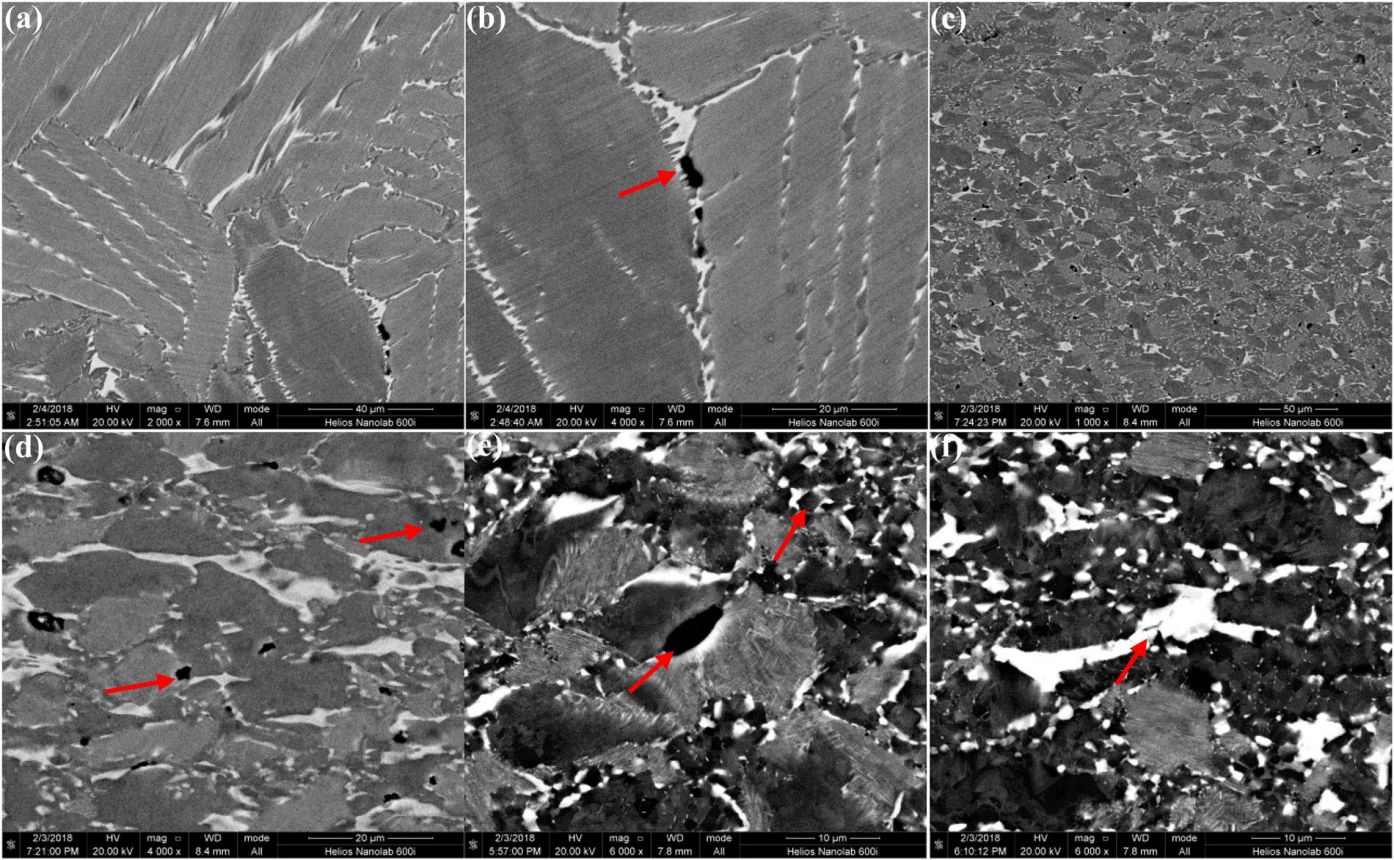
The SEM images of strain region on the tensile specimens of casted and forged Ti-43Al-6Nb-1Mo-1Cr alloys: (**a**,**b**) The weak boundaries in the casted alloys at 900 °C^23^; (**c**,**d**) The deformed DRXed grains in forged alloys at 900 °C; (**e**,**f**) The broken DRXed grains and cracks on boundaries in forged alloys at 800 °C.

The microstructure of strain region on the tensile specimens of casted and forged Ti-43Al-6Nb-1Mo-1Cr alloys are shown in Fig. 13. In Fig. 13(a,b)^23^, the holes form on the weak colonies boundaries and act as the sources of cracks formation in the casted alloys at 900 °C. In Fig. 13(c), the DRXed grains and colonies deform along the tensile direction in the forged alloys at 900 °C. When deformation exceeds the limit value, the DRXed grains break and the holes form on the boundaries at 900 °C, as exhibited in Fig. 13(d). In Fig. 13(e,f), however, the deformation of DRXed grains is less while less holes form on the boundaries at 800 °C. It means that the boundaries would be the weak positions and DRXed grains would deform better to support plastic deformation, with the temperature increasing.

**Figure 14:**
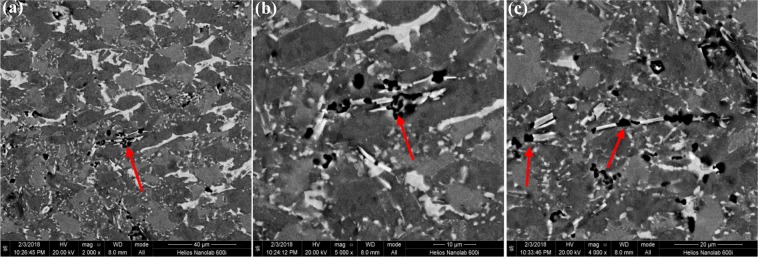
The SEM images of strain region on the tensile specimens of as-forged Ti-43Al-6Nb-1Mo-1Cr-0.6B alloys at 900 °C: (**a**) The holes on the grains boundaries; (**b**,**c**) The broken TiB phase.

The microstructure of strain region on the tensile specimens of the forged Ti-43Al-6Nb-1Mo-1Cr-0.6B alloys are shown in Fig. 14. In Fig. 14(a), holes form on the boundaries of DRXed grains and colonies while lamellae deform along the tensile direction. In this paper, TiB could also strengthen the forged alloys. In Fig. 14(b,c), dispersive TiB phase through α_2_/γ lamellae or around grain boundaries break, which indicates TiB phase could endure parts of stress and pin the boundaries or interface to hinder cracks formation. Li^34^ *et al*. have reported that TiB could cause many dislocation pile-ups to increase the UTS of TiAl alloys. Based on these two factors, boron addition could cause the strengthening in the forged alloys.

### The strengthening mechanisms

#### The strengthening mechanisms in the forged Ti-43Al-6Nb-1Mo-1Cr alloys

**Figure 15:**
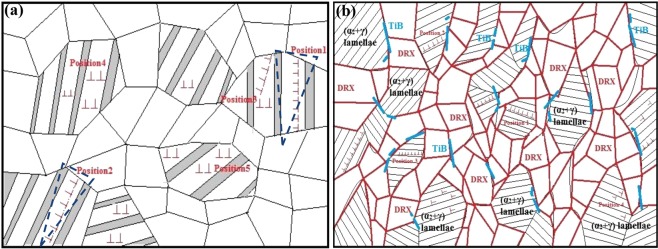
The strengthening and toughening mechanisms caused by multi steps forging process: (**a**) The forged Ti-43Al-6Nb-1Mo-1Cr alloys; (**b**) The forged Ti-43Al-6Nb-1Mo-1Cr-0.6B alloys.

The strengthening mechanism in Ti-43Al-6Nb-1Mo-1Cr alloys caused by the multi steps forging process is exhibited in Fig. 15(a). The uniform DRXed grains in alternating with finer (α_2_ + γ) lamellar colonies form a stable network structure in the forged microstructure. The strengthening mechanism: (i) The α_2_/γ lamellae obstacles hinder the dislocations movement to strengthen the alloys, as shown in Position 4 and Position 5. The finer grains increase the fraction and orientation diversity of α_2_/γ lamellae, which causes more dislocations pile-ups to strengthen the alloys; (ii) The isotropic grains obstacles cause dislocations pile-ups on the boundaries. Much finer grains increase the content dislocation blocks on the boundaries and to strengthen the alloys; (iii) The finer DRXed grains in alternating with (α_2_ + γ) lamellae form a stable triangular structure, as shown on Position 1 and Position 2. In Fig. 4(c), 60° and 90° angles occur between the α_2_/γ lamellae and the neighboring DRXed grains in the forged microstructure. It is all known that triangular structure is stable, while mangy dislocations would pile up in this type structure. Therefore, stress concentrations resulting from dislocation pile-ups are hard to break this structure. Additionally, this type of structure could also pin the α_2_/γ lamellar interfaces to hinder the cracks formation and growth along lamellar interfaces. Therefore, this normal triangular structure provides the stable and increased UTS for the forged alloys.

Based on above discussion, fine-grains strengthening, dislocation strengthening, grains boundaries and lamellar interfaces pinning would strengthen the forged alloys together. These mechanisms cause dramatically increased UTS at room temperature and 800 °C. With the temperature increasing, the combining power of grain boundaries decreasing would weaken the strengthening mechanisms. The finer grains deformation and boundaries slipping support the plastic deformation and increase elongation at higher temperatures.

The increased elongation of forged Ti-43Al-6Nb-1Mo-1Cr alloys could be explained as follow. The finer DRXed grains in alternating with α_2_/γ lamellae divide dislocations into different parts while the DRX process absorbs dislocations and reduces dislocation fraction. The separated dislocation blocks are difficult to reach the critical values of stress concentration and cracks formation, due to the separated and low energy. In this case, the separated dislocations would slip up continually until the stress concentration cause the cracks formation. The sustained dislocation movement supports the plastic deformation to increase the elongation^40^. In addition, the refined DRXed grains could deform to coordinate the plastic deformation of alloys at high temperatures. Huang^41^ *et al*. also proved that the refined DRXed grains could promote dislocation movement to support plastic deformation. Meanwhile, finer-grains could support plastic deformation better to increase the elongation.

#### The strengthening mechanisms in the forged Ti-43Al-6Nb-1Mo-1Cr-0.6 alloys

In the tensile curves, it can be found that the alloys with 0.6 at.% boron addition exhibit increased UTS, compared to the alloys without boron addition. The positive effect of boron addition on the high-temperature properties and the strengthening mechanism of casted alloys have been explored in ref.^34^. In this section, the effect of boron addition on the strengthening mechanisms of forged alloys should be clarified, as shown in Fig. 15(b). Relatively similar to the 3 types of strengthening mechanism in section 3.5.1, these strengthening mechanisms also occur in forged Ti-43Al-6Nb-1Mo-1Cr-0.6B alloys. Due to TiB formation around the grain boundaries, the boundaries movement during the forging process and annealing would be hinder while the grains growth could be limited. Combining with the promoted DRXed grains nucleation, the grains sizes in the forged Ti-43Al-6Nb-1Mo-1Cr-0.6B alloys were refined while the finer-grains strengthening was caused. The UTS of the forged Ti-43Al-6Nb-1Mo-1Cr-0.6B alloys was enhanced dramatically, compared to the forged Ti-43Al-6Nb-1Mo-1Cr alloys. With the temperature increasing, the combining power of grain boundaries would be weaker, as shown in Fig. 13(d–f). Dispersive TiB could pin and strengthen the boundaries which maintains strength at high temperatures. In addition, TiB obstacles could also hinder dislocation movement to strengthen the alloys through secondary phase strengthening at room and high temperatures. Therefore, the 0.6 at.% boron addition could increase the UTS of the forged alloys.

#### The decreased strength of forged alloys at 900 °C

By the multi steps forging process, the UTS and elongations of Ti-43Al-6Nb-1Mo-1Cr-(0,0.6)B alloys have been increased dramatically at room temperatures and 800 °C. With the temperature increasing to 900 °C, however, the elongation of forged Ti-43Al-6Nb-1Mo-1Cr-(0,0.6)B alloys increases, but the UTS decreases dramatically, compared to as-cast Ti-43Al-6Nb-1Mo-1Cr-0.6B alloys. The higher strengths of as-cast Ti-43Al-6Nb-1Mo-1Cr-0.6B alloys have been clarified in ref.^34^. The low strengths of forged Ti-43Al-6Nb-1Mo-1Cr-(0,0.6)B alloys at 900 °C should be discussed. In Table 1, the microstructure could be refined dramatically by the forging process. The combining power of grain boundaries would decrease dramatically, while grain boundaries slip easily with the temperature increasing to 900 °C, as shown in Fig. 13(c,d). The finer grains could deform easily while the increased grains boundaries could slip easily to support the plastic deformation better at 900 °C. Therefore, the refined microstructure increases the plasticity and reduces the deformation resistance dramatically. With the temperature increasing to 900 °C, the soft finer grains and weaker boundaries would weaken the strengthening from fine-grains, dislocations pile-ups and boundaries pinning effect. More dislocations pile-ups would be released in the soft matrix. All these factors lead to dramatically decreased UTS of forged alloys at 900 °C.

## Conclusions

The finer (α_2_ + γ) lamellae with DRXed grains and dispersive TiB form in the forged microstructure of Ti-43Al-6Nb-1Mo-1Cr-(0,0.6)B alloys.

(1) Multi-steps forging process could refine microstructure to increase the UTS of Ti-43Al-6Nb-1Mo-1Cr-(0,0.6)B alloys dramatically. The forged Ti-43Al-6Nb-1Mo-1Cr alloys exhibit the UTS as 645.65 ± 5.88 MPa at room temperature and 676.05 ± 11.37 MPa at 800 °C, while the grain size represents as 12.63 ± 3.77 μm. The dispersive TiB phase and much finer grains (average size is 10.32 ± 4.16 μm) could cause more dislocation pile-ups to enhance the UTS of the forged Ti-43Al-6Nb-1Mo-1Cr-0.6B alloys as 843.51 ± 2.67 MPa at room temperature and 729.00 ± 0.51 MPa at 800 °C.

(2) The strengthening mechanism in forged Ti-43Al-6Nb-1Mo-1Cr-(0,0.6)B alloys are caused by the finer microstructure: (i) More α_2_/γ lamellae obstacles for the dislocations movement; (ii) The finer-grains increase the content of dislocation pile-ups; (iii) The stable triquetrous structure consisting of DRXed grains and α_2_/γ lamellae hinder cracks formation and dislocation movement along α_2_/γ lamellae. (iiii) The dispersive TiB phase causes more dislocation pile-ups and pins grain boundaries and lamellar interfaces to strengthen the forged alloys containing 0.6 at.% boron.

(3) The increased elongation of forged Ti-43Al-6Nb-1Mo-1Cr-(0,0.6)B alloys at high-temperatures results from refined grains. The divided dislocations by refined grains delay the stress concentration and promote the sustained dislocation movement to support plastic deformation. With the temperature increasing, the finer grains could deform easily and the weaker grain boundaries would slip easily to support the plastic deformation.
